# Investigating molecular mechanisms of insecticide resistance in the Eastern Democratic Republic of the Congo

**DOI:** 10.1186/s12936-021-04002-8

**Published:** 2021-12-14

**Authors:** Janvier Bandibabone, Charles McLoughlin, Sévérin N’Do, Chimanuka Bantuzeko, Vital Byabushi, Muhigwa Jeanberckmans, Maite Guardiola, Bertin Zawadi, Abdoulaye Diabaté, Jorian Prudhomme, Thomas Walker, Louisa A. Messenger

**Affiliations:** 1 Laboratoire d’Entomologie Médicale et Parasitologie, Centre de Recherche en Sciences Naturelles (CRSN/Lwiro), Sud-Kivu, Democratic Republic of the Congo; 2 Department of Disease Control, Faculty of Infectious and Tropical Diseases, London School of Hygiene and Tropical Medicine, London, UK; 3 Médecins Sans Frontières (MSF) OCBA, Barcelona, Spain; 4 Université Officielle de Bukavu, Bukavu, Democratic Republic of the Congo; 5 Kibali Gold Mine, Haut-Uele, Democratic Republic of the Congo; 6 Université Nazi Boni (UNB), Bobo-Dioulasso, Burkina Faso; 7 UMR MIVEGEC (IRD-CNRS – Université de Montpellier), 911 Avenue Agropolis, 34394 Montpellier, France; 8 Institut de Recherche en Sciences de la Santé (IRSS)/Centre MURAZ, Bobo-Dioulasso, Burkina Faso

**Keywords:** *Anopheles gambiae*, *Anopheles funestus*, Insecticide resistance, *Plasmodium falciparum*, Democratic Republic of the Congo, Target site mutation, Metabolic resistance, Insecticide resistance surveillance

## Abstract

**Background:**

Malaria vector control in the Democratic Republic of the Congo is plagued by several major challenges, including inadequate infrastructure, lack of access to health care systems and preventative measures, and more recently the widespread emergence of insecticide resistance among *Anopheles* mosquitoes. Across 26 provinces, insecticide resistance has been reported from multiple sentinel sites. However, to date, investigation of molecular resistance mechanisms among *Anopheles* vector populations in DRC has been more limited.

**Methods:**

Adult *Anopheles gambiae* sensu lato (s.l.) and *Anopheles funestus* s.l. were collected from two sites in Sud-Kivu province and one site in Haut-Uélé province and PCR-screened for the presence of 11 resistance mutations, to provide additional information on frequency of resistance mechanisms in the eastern DRC, and to critically evaluate the utility of these markers for prospective country-wide resistance monitoring.

**Results:**

L1014F-*kdr* and L1014S-*kdr* were present in 75.9% and 56.7% of *An. gambiae* s.l. screened, respectively, with some individuals harbouring both resistant alleles. Across the three study sites, L43F-CYP4J5 allele frequency ranged from 0.42 to 0.52, with evidence for ongoing selection. G119S-*ace1* was also identified in all sites but at lower levels. A triple mutant haplotype (comprising the point mutation CYP6P4-I236M, the insertion of a partial Zanzibar-like transposable element and duplication of CYP6AA1) was present at high frequencies. In *An. funestus* s.l.* cis*-regulatory polymorphisms in CYP6P9a and CYP6P9b were detected, with allele frequencies ranging from 0.82 to 0.98 and 0.65 to 0.83, respectively.

**Conclusions:**

This study screened the most up-to-date panel of DNA-based resistance markers in *An. gambiae* s.l. and *An. funestus* s.l. from the eastern DRC, where resistance data is lacking. Several new candidate markers (CYP4J5, G119S-*ace1*, the triple mutant, CYP6P9a and CYP6P9b) were identified, which are diagnostic of resistance to major insecticide classes, and warrant future, larger-scale monitoring in the DRC to inform vector control decisions by the National Malaria Control Programme.

**Supplementary Information:**

The online version contains supplementary material available at 10.1186/s12936-021-04002-8.

## Background

Malaria is a significant public health concern in the Democratic Republic of the Congo (DRC), where it is currently responsible for 12% and 11% of global malaria cases and deaths, respectively [1]. In the DRC, approximately 60% of the population reside in zones with an average *Plasmodium falciparum* prevalence above 50% [2]; malaria is the leading cause of medical consultations, hospitalizations and death [3], accounting for 44% of all outpatient visits and 22% of deaths in children under 5 years old [4]. Malaria vector control in the DRC relies on universal coverage of insecticide-treated nets (ITNs), via mass campaigns, community-based top-ups to maintain high coverage, and continuous distributions through routine antenatal care (ANC) and child immunization services by the national expanded programme on immunization (EPI) [5]. Small-scale indoor residual spraying (IRS) is also undertaken by private enterprises (usually mining operations) in focal areas [5]. Between 2011 and 2018, an estimated 134.8 million pyrethroid-treated ITNs were distributed nationwide through such mechanisms [6]. However, recent estimates of net access and use across the country remain low, with the proportion of households with at least one ITN for every two people declining from 47% to 2013/14 to 44% in 2017/18, and the proportion of children under five years old reported sleeping under an ITN the previous night also decreasing from 56% to 2013/14 to 51% in 2017/18 [7].

Malaria control in the DRC is plagued by several major challenges, including poor transportation and communication infrastructure, a majority rural population, high poverty, political and socio-economic instability, lack of access to health care systems and preventative measures for at-risk populations, and more recently the widespread emergence of insecticide resistance among *Anopheles* vector populations. Across 26 provinces in the DRC, insecticide resistance in *An. gambiae* sensu lato (s.l.) and *An. funestus* s.l. has been reported from multiple sentinel sites [8], including reduced susceptibility to DDT, deltamethrin and permethrin in Kwilu [9], Nord-Ubangi [10] and Sud-Kivu provinces [11], and to deltamethrin and DDT in Haut-Uélé province [12]. In Kinshasa, high intensity pyrethroid and organochlorine resistance has been observed, with *An. gambiae* populations displaying low mortality after 6 h of exposure to DDT and permethrin [13], and significant proportions of vectors are capable of surviving exposure to five and ten times the diagnostic doses of alpha-cypermethrin, deltamethrin and permethrin [14]. Importantly, nationwide Demographic and Health surveys (DHS) in the DRC indicate higher levels of protection with ITNs containing deltamethrin, compared to permethrin, suggesting a partial influence of insecticide resistance on vector control intervention efficacy [15]. By comparison to pyrethroids, resistance profiles to other insecticide classes (e.g. organophosphates and carbamates) have been less clearly established, restricting the ability of the national malaria control programme (NMCP) to make evidence-based decisions for resistance management.

To date, the investigation of insecticide resistance mechanisms among *Anopheles* vector populations in DRC has been limited (summarized in Additional file 1: Table S1). L1014F- and L1014S-*kdr* mutations are found at variable frequencies in *An. gambiae* across the country, with the former predominating in western and central provinces (Additional file 1: Table S1); a proportion of individuals have also been documented harbouring both L1014F and L1014S alleles. N1575Y is present at very low prevalence in Nord-Ubangi province [10], while other commonly described mutations, such as G119S-*ace1*, have not been detected in the DRC (Additional file 1: Table S1). Among local *An. funestus*, overexpression of key detoxification enzymes (including CYP6P9a, CYP6P9b, CYP6M7, CYP6P4a, CYP6P4b and GSTE2) has been identified in pyrethroid resistant field populations [12, 13] (Additional file 1: Table S1). Furthermore, increased mortality following pre-exposure of resistant *An. gambiae* to the synergist piperonyl butoxide (PBO) before pyrethroid bioassays, also indicates a role for metabolic resistance mechanisms in this species complex in the DRC [8, 10], supported by reports of overexpression of CYP6M2 and CYP6P1 [12].

While recent next-generation sequencing initiatives have characterised substantial genetic diversity within natural *Anopheles* populations, concerns have been raised for the rapid evolution and spread of novel insecticide resistance mechanisms [16, 17]. However, real-time tracking of resistance mechanisms in the field, especially the identification of diagnostic markers that are predictive of vector control intervention failure [18, 19], is still lacking. As an intermediate step for future insecticide resistance monitoring efforts in the DRC, this study assessed *P. falciparum* infection prevalence and the frequency of 11 published insecticide resistance mutations among populations of *An. gambiae* s.l. and *An. funestus* s.l., collected from three areas of pyrethroid resistance in the eastern DRC.

## Methods

### Mosquito collections and species identification

**Fig. 1:**
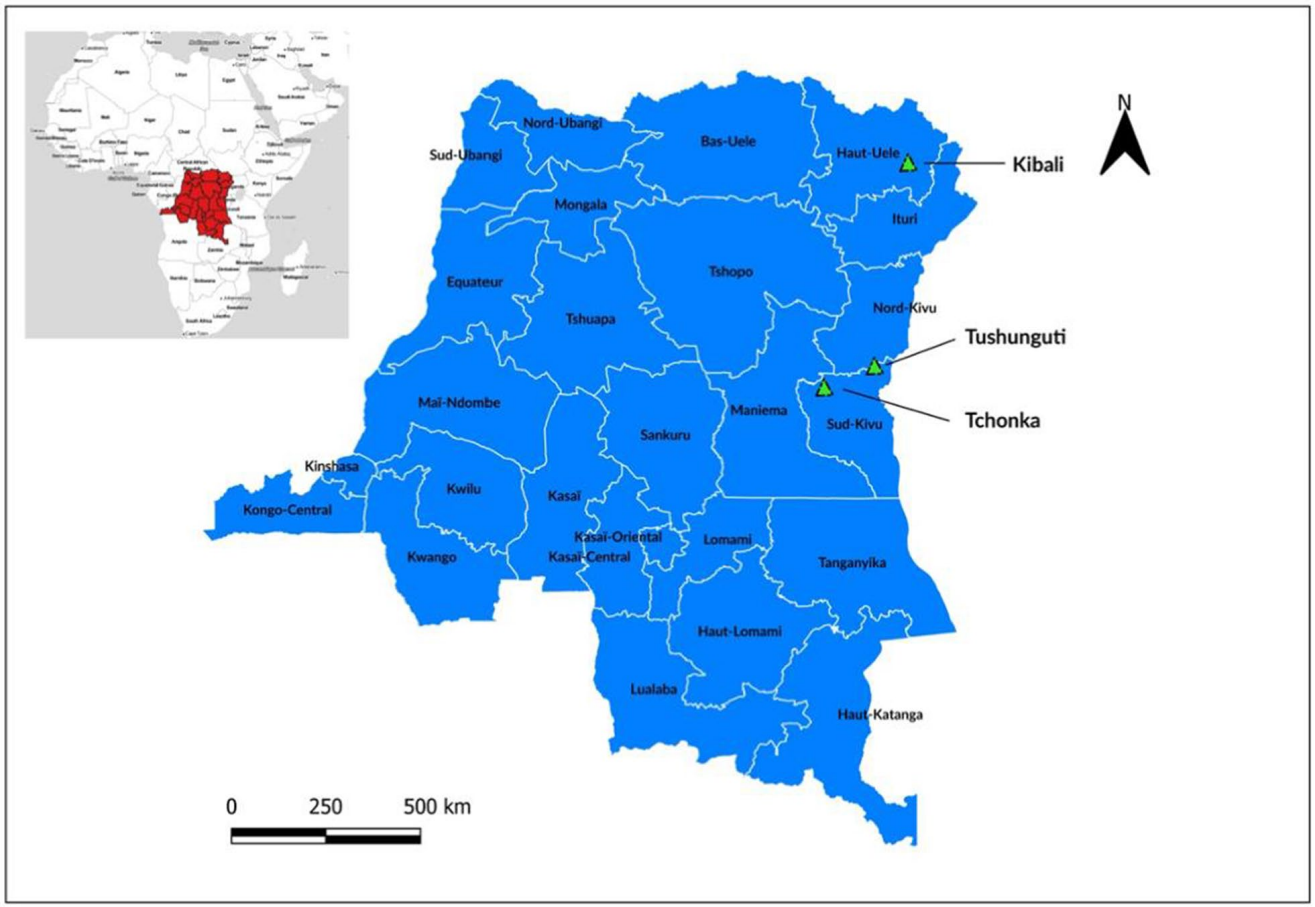
Map of study sites in Sud-Kivu (Tchonka, and Tushunguti) and Haut-Uélé (Kibali) provinces, in the Democratic Republic of the Congo

Adult *Anopheles* were collected from households in two sites in Sud-Kivu province (Tchonka; 2° 19′ 18″ S, 27° 32′ 24″ E and Tushunguti; 1° 48′ 19″ S, 28° 45′ 00.5″ E) and one site in Haut-Uélé province (Kibali; 3° 6′ 59″ N, 29° 35′  8″ E) using Centers for Disease Control (CDC) light traps during the rainy season in Tchonka (100 houses: April-June 2018), Tushunguti (50 houses: December 2017-February 2018) and Kibali (25 houses: June 2019) (Fig. 1). Mosquitoes were identified morphologically as members of the *An. gambiae* s.l. or *An. funestus* s.l. complexes [20].

Individual mosquitoes were homogenized in a Qiagen TissueLyser II with sterilized 5 mm stainless steel beads for 5 min at 30 Hz and incubated overnight at 56 °C. DNA was extracted using a Qiagen DNeasy 96 blood and tissue kit (Qiagen, UK), according to the manufacturer’s protocol. A subset of mosquitoes, morphologically classified as *An. gambiae* s.l. (n = 24) or *An. funestus* s.l. (n = 16), were further identified to species-level by PCR [21, 22]. A total of 163 *An. funestus* s.l. from Tchonka (n = 133) and Tushunguti (n = 30) and 192 *An. gambiae* s.l. from Tchonka (n = 131), Tushunguti (n = 32) and Kibali (n = 29) were used for insecticide resistance mutation analyses.

### *Plasmodium falciparum* screening

Individual mosquitoes (n = 355) were screened for the presence of *P. falciparum* using a SYBR green real-time assay targeting the parasite cytochrome c oxidase subunit 1 (*cox1*) mitochondrial gene (present in all stages of the *P. falciparum* life cycle) [23].

### *Anopheles gambiae* s.l. target site mutation screening

Mosquito individuals morphologically identified as *An. gambiae* s.l. (n = 192) were screened for eight mutations: L1014S-*kdr*, L1014F-*kdr*, G119S-*ace1*, N1575Y, L43F-CYP4J5, CYP6P4 (I236M), Zanzibar-like transposable element (TE) and CYP6AA1 duplication.

PCR reaction primers, probes and conditions for all assays are detailed in Table 1.

**Table 1:** PCR primers, probes and reaction conditions

	Primers and probes (concentrations)	5′ modifications	Sequences	Reaction conditions	References
*An. gambiae* s.l. mutation					
L1014F-*kdr*	IPCF (2.5 pmol/µL) AltRev (2.5 pmol/µL) WT (25 pmol/µL) West (8 pmol/µL)		GATAATGTGGATAGATTCCCCGACCATG TGCCGTTGGTGCAGACAAGGATG GGTCCATGTTAATTTGCATTACTTACGAATA CTTGGCCACTGTAGTGATAGGAAATGTT	1 cycle: 5 min at 95 °C 35 cycles: 30 s at 95 °C, 30 s at 59 °C, 30 s at 72 °C 1 cycle: 5 min at 72 °C	[25]
L1014S-*kdr*	IPCF (2.5 pmol/µL) AltRev (2.5 pmol/µL) WT (5 pmol/µL) East (2.5 pmol/µL)		GATAATGTGGATAGATTCCCCGACCATG TGCCGTTGGTGCAGACAAGGATG GGTCCATGTTAATTTGCATTACTTACGAATA CTTGGCCACTGTAGTGATAGGAAAATC	1 cycle: 5 min at 95 °C 35 cycles: 30 s at 95 °C, 30 s at 57 °C, 30 s at 72 °C 1 cycle: 5 min at 72 °C	[25]
N1575Y	Forward (1 µM) Reverse (1 µM) N (0.5 µM) Y (0.5 µM)	HEX FAM	TGGATCGCTAGAAATGTTCATGACA CGAGGAATTGCCTTTAGAGGTTTCT ATTTTTTTCATTGCATTATAGTAC TTTTTCATTGCATAATAGTAC	1 cycle: 15 min at 95 °C 40 cycles: 15 s at 94 °C, 1 min at 60 °C	[24]
G119S-*ace1*	Forward (0.8 µM) Reverse (0.8 µM) G (0.2 µM) S (0.2 µM)	HEX FAM	GGCCGTCATGCTGTGGAT GCGGTGCCGGAGTAGA TTCGGCGGCGGCT TTCGGCGGCAGCT	1 cycle: 10 min at 95 °C 40 cycles: 10 s at 95 °C, 35 s at 60 °C	[25]
L43F-CYP4J5	Forward (1 µM) Reverse (1 µM) Probe 1 (0.5 µM) Probe 2 (0.5 µM)	FAM HEX	AGCCTGCGCGTGTGATA CTTCTTCTCCTGTGGTTCGTTTG TTGCCGGAAGGCAGT TTGCCGGAGGGCAGT	1 cycle: 10 min at 95 °C 40 cycles: 15 s at 92 °C, 1 min at 60 °C	[26]
CYP6P4-I236M	CYP6P4_I236M_Forward (0.35 µM) CYP6P4_I236M_Reverse (0.35 µM) CYP6P4_I-Wild (0.2 µM) CYP6P4_M-Mutant (0.2 µM)	FAM HEX	AGTTTATGTTTGCGACCACGTT TCCACCGTCTCGCGCACAAC TTC+ATGC+C+G+ATGC TTC+ATGC+C+C+ATGC	1 cycle: 3 min at 95 °C 20 cycles: 15 s at 95 °C, 30 s at 66 °C 23 cycles: 10 s at 95 °C, 20 s at 58 °C, 11 s at 72 °C	[27]
Zanzibar-like TE	ZZB_Flank_A (0.35 µM) ZZB_Flank_B (0.35 µM) ZZB_Int_A (0.35 µM) ZZB_Mutant (0.2 µM) ZZB_WT (0.2 µM)	FAM HEX	CAAAATCAATGKCACRGAGC CGCTACAATGAAKGGAAAGTC CATTACATGGCGACCGTACCT A+C+ATTA+CA+CTTTGT+CA+GTA GATG+TT+CTTTK+T+CA+G+TATT	1 cycle: 3 min at 95 °C 40 cycles: 10 s at 95 °C, 20 s at 58 °C, 11 s at 72 °C	[27]
CYP6AA1 duplication	AA1_Dup1_ins1 (0.35 µM) AA1_Dup1_outs (0.35 µM) AA1_Dup1_ins2 (0.35 µM) AA1_Dup1_outs (0.2 µM) AA1_Dup1_ins (0.2 µM) AA1_Dup1_junct (0.2 µM)	Cy5 HEX FAM	CAGTGCGGTACGCTCGTTAA GGATCGGTTTACAGCGGACG CATCACCTGTGCTCGCAARTT CCAT+CA+C+CGAA+CG AGAA+CCTGCA+C+CAA A+CA+AT+TAATT+G+CAT+CGG	1 cycle: 3 min at 95 °C 40 cycles: 10 s at 95 °C, 20 s at 57 °C, 15 s at 72 °C	[27]
2La inversion	23A2 (25 pmol/µL) 27A2 (25 pmol/µL) DPCross 52 L+ (25 pmol/µL)		CTCGAAGGGACAGCGAATTA ACACATGCTCCTTGTGAACG GGTATTTCTGGTCACTCTGTTGG	1 cycle: 2 min at 94 °C 35 cycles: 30 s at 94 °C, 30 s at 60 °C, 45 s at 72 °C 1 cycle: 10 min at 72 °C	[25]
*An. funestus* s.l. mutation					
L119F-GSTe2	Forward (1 µM) Reverse (1 µM) L119 (0.5 µM) 119 F (0.5 µM)	HEX FAM	AACAATTTTTCATTTCTTATTCTCATTTAC CGACTCGATCTTCGGGAATGTC AGGAGCGTATTCTTTTCTAC AGGAGCGTATTTTTTTCTA	1 cycle: 10 min at 95 °C 40 cycles: 15 s at 92 °C and 1 min at 60 °C	[28]
CYP6P9a	Forward (1 µM) Reverse (1 µM)		TCCCGAAATACAGCCTTTCAG ATTGGTGCCATCGCTAGAAG	1 cycle: 95 °C for 3 min 40 cycles: 94 °C for 30 s, 55 °C for 30 s, 72 °C for 1 min 1 cycle: 72 °C for 10 min	[18]
CYP6P9b	Forward (1 µM) Reverse (1 µM)		CCCCCACAGGTGGTAACTATCTGAA TTATCCGTAACTCAATAGCGATG	1 cycle: 95 °C for 3 min 40 cycles: 94 °C for 30 s, 58 °C for 30 s, 72 °C for 1 min 1 cycle: 72 °C for 10 min	[19]

For L1014F-*kdr*, amplifications were performed in 25 µL reactions containing 2 µL template DNA, 1 µL IPCF, 1 µL AltRev, 1 µL WT, 3 µL West, 4.5 µL H_2_O, and 12.5 µL 2× Hot Start Taq PCR Master Mix (New England Biolabs, UK). For L1014S-*kdr*, amplifications were performed in 25 µL reactions containing 2 µL template DNA, 2 µL IPCF, 2 µL AltRev, 2 µL WT, 2 µL East, 2.5 µL H_2_O, and 12.5 µL 2× Hot Start Taq PCR Master Mix (New England Biolabs, UK). PCR products were separated in 2% E-Gel^TM^ agarose gels with SYBR Safe (Invitrogen, UK). A control band at 314 base pairs (bp) indicated a successful reaction, a band at 214 bp indicated the susceptible wild type allele, and a band at 156 bp indicated the resistant allele. No template controls (NTCs) were run in parallel for all assays.

For N1575Y, PCR reactions were prepared with 10 µL of 2× QuantiTect^TM^ Probe PCR master mix (Qiagen, UK), the primers and probes listed in Table 1, and 2 µL template DNA for a final reaction volume of 20 µL [24]. Positive controls from gDNA extracted from known *An. gambiae* sensu stricto (s.s.) with and without the N1575Y mutation were included in each run, alongside NTCs.

**Table 2:** L1014F and L1014S allele frequencies among *An. gambiae* s.l. from three study sites in East DRC

Study site (Province)	# Mosquitoes tested^a^	Homozygote L1014F/L1014F (RR)	Homozygous L1014S/L1014S (RR)	Heterozygous^b^ L1014F/L1014S (RR)	Homozygous wild type (SS)	Allele frequency			χ^2^ test	*p*-value
						L1014F (R)	L1014S (R)	L1014 (S)		
Tchonka (Sud-Kivu)	126	66	4	56	0	0.75	0.25	–	–	–
Tushunguti (Sud-Kivu)	32	4	9	14	5	0.34	0.5	0.16	0.14	0.71
Kibali (Haut-Uélé)	29	1	22	1	5	0.05	0.78	0.17	22.86	< 0.0001

^a^Sample numbers adjusted to reflect non-amplifiers per assay

^b^No heterozygous individuals for either L1014F or L1014S alone were detected

For G119S-*ace1*, PCR reactions were prepared with 5 µL 2× PrimeTime® Gene Expression Master Mix (Integrated DNA Technologies, UK), the primers and probes listed in Table 1, and 2 µL template DNA for a final reaction volume of 10 µL [25]. Positive controls from gDNA extracted from known *An. gambiae* s.s. with and without the G119S-*ace1* mutation were included in each run, alongside NTCs.

For L43F-CYP4J5, PCR reactions were prepared with 10 µL of 2× QuantiTect^TM^ Probe PCR master mix (Qiagen, UK), the primers and probes listed in Table 1, and 2 µL template DNA for a final reaction volume of 20 µL [26].

Three recently designed, independent, locked-nucleic acid (LNA) probe-based PCR assays were used to genotype the point mutation CYP6P4-I236M, the insertion of the partial Zanzibar-like TE and the duplication of CYP6AA1 [27]. For each assay, PCR reactions were prepared with 5 µL 2× PrimeTime® Gene Expression Master Mix (Integrated DNA Technologies, UK), the primers and probes listed in Table 1, and 2 µL template DNA for a final reaction volume of 10 µL.

For the 2La inversion region in *An. gambiae* s.l. which contains L43F-CYP4J5 [26], amplifications were performed in 25 µL reactions containing 12.5 µL 2× Hot Start Taq PCR Master Mix (New England Biolabs, UK), the primers listed in Table 1, and 2 µL template DNA [25]. PCR products were separated in 2% E-Gel^TM^ agarose gels with SYBR Safe (Invitrogen, UK). A band at 492 bp or 207 bp indicated the 2La or 2L+^a^ arrangement, respectively.

### *Anopheles funestus* s.l. target site mutation screening

Mosquito individuals morphologically identified as *An. funestus* s.l. (n = 163) were screened for three metabolic mutations: L119F-GSTe2, CYP6P9a and CYP6P9b.

For L119F-GSTe2, PCR reactions were prepared with 10 µL of 2× QuantiTect^TM^ Probe PCR master mix (Qiagen, UK), the primers and probes listed in Table 1, and 2 µL template DNA for a final reaction volume of 20 µL [28].

For CYP6P9a, PCR reactions were performed in a final volume of 25 µL, containing 2× Hot Start Taq PCR Master Mix (New England Biolabs, UK), the primers listed in Table 1, and 2 µL template DNA [18]. Ten microlitres of each PCR product were digested by adding 1 µL 10× buffer TaqI, 0.2 µL TaqI restriction enzyme (Thermo Scientific, UK) and 3.8 µL of H_2_O. Digests were incubated at 65 °C for 4 h. Digested products were separated in 2% E-Gel^TM^ agarose gels with SYBR Safe (Invitrogen, UK). A band at 450 bp indicated the susceptible wild type allele, a band at 350 bp and 100 bp indicated the resistant allele.

For CYP6P9b, PCR reactions were performed in a final volume of 25 µL, containing 2× Hot Start Taq PCR Master Mix (New England Biolabs, UK), the primers listed in Table 1, and 2 µL template DNA [19]. Ten microlitres of each PCR product were  digested by adding 1 µL CutSmart buffer, 0.2 µL Tsp45I restriction enzyme (New England Biolabs, UK) and 3.8 µL of H_2_O. Digests were incubated at 65 °C for 4 h. Digested products were separated in 2% E-Gel^TM^ agarose gels with SYBR Safe (Invitrogen, UK). A band at 550 bp indicated the resistant allele and two bands at 400 bp and 150 bp indicated the susceptible wild type allele.

For both CYP6P9a (n = 8) and CYP6P9b (n = 8), PCR-RFLP results for a subset of resistant and susceptible individuals were confirmed by sequencing as previously described [29]. In brief, PCR products used in the enzyme digests were submitted to Source BioScience (Source BioScience Plc, Nottingham, UK) for PCR reaction clean-up, followed by chain termination sequencing. Sequencing analysis was conducted in Geneious Prime®2021.1.1.

### Data analysis

Stratagene MxPro qPCR software (Agilent Technologies, UK) was used to produce standard curves for genotypic analysis. All statistical analyses were conducted in Stata/SE 17.0, including Pearson’s Chi-squared test to investigate deviations from Hardy–Weinberg equilibrium and associations between study site, presence/absence of resistance mutation and *P. falciparum* infection prevalence. Analysis of the triple LNA PCR assay to detect the point mutation CYP6P4-I236M, the insertion of the partial Zanzibar-like TE and the duplication of CYP6AA1, was conducted according to Njoroge et al. [27]. Heterozygotes and homozygotes for the CYP6AA1 duplication were differentiated by analysis of the ratio of the HEX, FAM and Cy5 Ct values: 2*Cy5-(FAM+HEX); ratio values were then arranged in ascending order, plotted on a line graph and heterozygotes and homozygotes differentiated by a change in the line gradient.

## Results

### Mosquito species identification and *P. falciparum* screening

A subset of eight individual *An. gambiae* s.l. each from Tchonka, Tushunguti and Kibali and *An. funestus* s.l. from Tchonka and Tushunguti were identified to species-level by PCR; all were determined to be *An. gambiae* s.s. (24/24) and *An. funestus* s.s. (16/16), respectively. *P. falciparum* infection rate (indicative of any parasite lifecycle stage) was 11.04% (18/163) and 10.94%  (21/192) among *An. funestus* s.l. and *An. gambiae* s.l., respectively. By study site, *P. falciparum* infection rate for *An. funestus* s.l. was not significantly different between Tchonka (12.03%; 16/133) and Tushunguti (6.7%; 2/30) (χ^2^ = 0.717, *p *= 0.397), nor for *An. gambiae* s.l. (13.0%; 17/131 in Tchonka, 6.25%; 2/32 in Tushunguti and 6.9%; 2/29 in Kibali; χ^2^ = 1.77, *p *= 0.413).

### *Anopheles gambiae* s.l. target site mutation screening

Mosquito individuals morphologically identified as *An. gambiae* s.l. were screened for the presence of eight mutations (Tables 2 and 3). L1014F-*kdr* was present in 75.9% (142/187) of *An. gambiae* s.l. screened; 50% (71/142) were homozygous L1014F/L1014F and 50% were heterozygous L1014F/L1014S (Table 2). L1014S-*kdr* was present in 56.7% (106/187) of *An. gambiae* s.l. tested; 33.0% (35/106) were homozygous L1014S/L1014S and 67.0% (71/106) were heterozygous L1014F/L1014S (Table 2). No individuals were heterozygous for either L1014F or L1014S mutation alone. By study site there was a clear predominance of L1014F/L1014F (52.4%; 66/126) and L1014F/L1014S (44.4%; 56/126) in Tchonka, and L1014S/L1014S in Kibali (75.9%; 22/29) (Table 2). In Kibali, there was evidence for selection acting on this locus (χ^2^ = 22.86, *p *< 0.0001). N1575Y was not detected in any individual tested (190/190).

**Table 3:** *An. gambiae* s.l. target site allele frequencies from three study sites in East DRC

Resistance mutation	Study site (Province)	# Mosquitoes tested^a^	Homozygote mutation (RR)	Heterozygote mutation (RS)	Homozygote wild type (SS)	Allele frequency			χ^2^ test	*p*-value
						R	S			
N1575Y	Tchonka (Sud-Kivu)	129	0	0	129	0	1.0		–	–
	Tushunguti (Sud-Kivu)	32	0	0	32	0	1.0		–	–
	Kibali (Haut-Uélé)	29	0	0	29	0	1.0		–	–
L43F-CYP4J5	Tchonka (Sud-Kivu)	126	5	95	26	0.42	0.58		38.26	< 0.0001
	Tushunguti (Sud-Kivu)	32	1	31	0	0.52	0.48		28.24	< 0.0001
	Kibali (Haut-Uélé)	28	0	24	4	0.43	0.57		15.75	< 0.0001
G119S-*ace1*	Tchonka (Sud-Kivu)	131	1	41	89	0.16	0.84		2.59	0.11
	Tushunguti (Sud-Kivu)	32	0	12	20	0.19	0.81		1.70	0.19
	Kibali (Haut-Uélé)	28	1	4	23	0.11	0.89		1.80	0.18

^a^Sample numbers adjusted to reflect non-amplifiers per assay

L43F-CYP4J5 was identified in 83.9% (156/186) of *An. gambiae* s.l. tested; 3.8% (6/156) were homozygous and 96.2% (150/156) were heterozygous (Table 3). Across the three study sites, L43F-CYP4J5 allele frequency ranged from 0.42 to 0.52, with evidence for significant deviations from Hardy–Weinberg equilibrium (χ^2^ = 38.26, 28.24 and 15.75 for Tchonka, Tushunguti and Kibali, respectively; *p *< 0.0001 for all). Overall, 91.8% (168/183) of *An. gambiae* s.l. tested harboured the 2L+^a^ inversion; 42.1% (77/183) were 2L+^a^/+^a^ homozygous and 49.7% (91/183) were 2La+^a^ heterozygous (Table 4). Across the three study sites, evidence for ongoing selection for the 2La inversion was observed in Tchonka only (χ^2^ = 15.24, *p *< 0.0001).

**Table 4:** *An. gambiae* s.l. 2La karyotypes from three study sites in East DRC

Study site (Province)	# Mosquitoes tested^a^	2La/a	2La+^a^ hybrid	2L+^a^/+^a^	χ^2^ test	*p*-value
Tchonka (Sud-Kivu)	125	1	68	56	15.24	< 0.0001
Tushunguti (Sud-Kivu)	30	9	10	11	3.27	0.07
Kibali (Haut-Uélé)	28	5	13	10	0.05	0.83

^a^Sample numbers adjusted to reflect non-amplifiers per assay

G119S-*ace1* was detected in 30.9% (59/191) of *An. gambiae* s.l. tested; 3.4% (2/59) were homozygous and 96.6% (57/59) were heterozygous (Table 3). There was no evidence for ongoing selection for G119S-*ace1* in any study site, with the resistant allele frequency ranging from 0.11 to 0.19 (Table 3).

For *An. gambiae* s.l., there was no significant association with presence of any resistant allele and *P. falciparum* infection for L1014F-*kdr* (χ^2^ = 1.15, *p *= 0.283), L1014S-*kdr* (χ^2^ = 1.75, *p *= 0.186), L43F-CYP4J5 (χ^2^ = 1.30, *p *= 0.254) or G119S-*ace1* (χ^2^ = 3.05, *p *= 0.081); nor 2L^+a^ inversion and *P. falciparum* infection (χ^2^ = 0.32, *p *= 0.573).

The triple LNA PCR assay, used to genotype the point mutation CYP6P4-I236M, the insertion of the partial Zanzibar-like TE and the duplication of CYP6AA1, identified high frequencies of the triple homozygote mutant in all study sites (Table 5). Furthermore, the double mutant CYP6P4-I236M-ZZB-TE was detected in nine *An. gambiae* s.l. from Tchonka.

**Table 5:** *An. gambiae* s.l. triple mutant genotype (CYP6P4-I236M-Zanzibar-like TE-CYP6AA1 duplication) frequencies from three study sites in East DRC

Study site (Province)	Homozygote CYP6P4-I236M-ZZB-TE wild type	Double CYP6P4-I236M-ZZB-TE mutant	Heterozygote CYP6P4-I236M-ZZB-TE-CYP6AA1 duplication (triple mutant)	Homozygote CYP6P4-I236M-ZZB-TE-CYP6AA1 duplication (triple mutant)	CYP6P4-I236M-ZZB-TE-CYP6AA1 duplication (triple mutant) frequency
Tchonka (Sud-Kivu)	0	9	4	33	0.76
Tushunguti (Sud-Kivu)	1	0	0	1	0.5
Kibali (Haut-Uélé)	0	0	1	14	0.97

### *Anopheles funestus* s.l. metabolic  mutation screening

Mosquito individuals morphologically identified as *An. funestus* s.l. were screened for three mutations in metabolic genes (Table 6). L119F-GSTe2 was not detected in any individual tested (Table 6). CYP6P9a was present in 100% of *An. funestus* s.l. tested (152/152); 67.8% (103/152) were homozygous and 32.2% (49/152) were heterozygous (Table 6). In Tchonka CYP6P9a allele frequency was 0.82, with significant deviations from Hardy–Weinberg equilibrium (χ^2^ = 6.59; *p *= 0.01); no evidence for ongoing selection was observed in Tushunguti (Table 6). CYP6P9b was present in 94.9% of *An. funestus* s.l. screened (131/138); 70.2% (92/131) were homozygous and 29.8% (39/131) were heterozygous (Table 6). In Tushunguti CYP6P9b allele frequency was 0.65, with significant deviations from Hardy–Weinberg equilibrium (χ^2^ = 4.0; *p *= 0.05); no evidence for ongoing selection was observed in Tchonka (Table 6). Presence of the CYP6P9b resistant allele was significantly associated with *P. falciparum* infection (χ^2^ = 7.03, *p *= 0.008), while presence of the CYP6P9a resistant allele was not (χ^2^ = 1.39, *p *= 0.238).

**Table 6:** *An. funestus* s.l. metabolic  allele frequencies from two study sites in East DRC

Resistance mutation	Study site	# Mosquitoes tested^a^	Homozygote mutation (RR)	Heterozygote mutation (RS)	Homozygote wild type (SS)	Allele frequency			χ^2^ test	*p*-value
						R	S			
L119F-GSTe2	Tchonka	126	0	0	126	0	1.0		–	–
	Tushunguti	27	0	0	27	0	1.0		–	–
CYP6P9a	Tchonka	131	83	48	0	0.82	0.18		6.59	0.01
	Tushunguti	21	20	1	0	0.98	0.02		0.01	0.91
CYP6P9b	Tchonka	121	83	35	3	0.83	0.17		0.09	0.76
	Tushunguti	17	9	4	4	0.65	0.35		4.0	0.05

^a^Sample numbers adjusted to reflect non-amplifiers per assay

## Discussion

By comparison to neighbouring malaria-endemic countries, there is a considerable paucity of available molecular insecticide resistance data in the DRC. This study assessed the frequency of 11 resistance mutations among field populations of *An. gambiae* s.l. and *An. funestus* s.l., to provide additional information on resistance mechanisms in the eastern DRC, and to critically evaluate the utility of these markers for prospective country-wide resistance monitoring.

Because the NMCP vector control strategy relies almost exclusively on universal coverage of ITNs, high levels of pyrethroid resistance and cross-resistance to DDT are widespread, with some evidence for increasing pyrethroid resistance intensity following distribution of ITNs in parts of Kinshasa province [14]. Unsurprisingly, the pyrethroid-associated L1014F-*kdr* and L1014S-*kdr* mutations in the voltage-gated sodium channel (VGSC) are present in *An. gambiae* s.l. across the country and can be found in high frequencies in some locales [11, 14]. Prior to this study, molecular resistance monitoring had been undertaken in Kibali in 2011–2012, reporting a moderate prevalence of L1014S-*kdr* (0.61) and lower levels of L1014F-*kdr* (0.1) and L1014F/S-*kdr* (0.26) [12] (Additional file 1: Table S1). The L1014S-*kdr* allele frequency of 0.78 in this dataset from 2017-2019 supports these previous surveys, with evidence for ongoing selection of this allele. One additional study has been performed in Tchonka from April–November, 2018 [11] indicating almost complete fixation of L1014F-*kdr* (0.98), which was also consistent with this study’s observations of slightly lower frequencies of this mutation (0.75), which may have risen over time under selection. The predictive association between L1014F-*kdr* and L1014S-*kdr* and resistance phenotype (i.e. survival or death following an insecticide bioassay or exposure to a vector control intervention) is not absolute [30], but both mutations have been proposed to play a larger contributing role in resistance to type I (permethrin) versus type II (alpha-cypermethrin and deltamethrin) pyrethroids [31], which broadly aligns with observations of lower permethrin susceptibility, compared to deltamethrin or alpha-cypermethrin, in some local vector populations [10, 11, 14, 32]; it is also important to note that differences in discriminating concentrations of pyrethroids used for resistance monitoring and other coinciding resistance mechanisms may also explain some of these discrepancies [33]. This study also demonstrated a proportion of *An. gambiae* s.l. individuals with both L1014F-*kdr* and L1014S-*kdr* mutations co-occurring in Sud-Kivu province. This phenomenon has also been observed in Kinshasa [14] and Nord-Ubangi provinces [10], as well as in other areas of East and West Africa [34, 35]. While the biological implications of harbouring both resistant alleles are unclear, it adds an additional complication to routine genotypic surveillance and supports the use of newly-developed single detection assays [10]. A second mutation in the VGSC, N1575Y, located downstream of L1014F-*kdr*, can have a synergistic effect on pyrethroid and DDT resistance [24, 36], but was not observed in any *An. gambiae* s.l. screened. It has only been reported once previously in the DRC, at very low frequencies in *An. gambiae* s.s. and *Anopheles coluzzii* from Nord-Ubangi [10]. The recent characterization of 20 additional non-synonymous nucleotide substitutions in the VGSC has revealed that the molecular basis of target-site pyrethroid resistance in malaria vectors may be more complex than previously thought [37]. This is of particular relevance in the DRC where high genetic diversity in *kdr* haplotypes has been described, suggesting that these resistance alleles may have either originated in central Africa and spread across the continent or converged in the DRC and persisted without replacement [38].

In all three study sites, a non-synonymous substitution in CYP4J5 was identified at moderate levels (0.42–0.52), for the first time in the DRC. The point mutation used in this assay is in tight linkage disequilibrium (LD) with the L43F-CYP4J5 variant, which has previously been associated with resistance to lambda-cyhalothrin in *An. gambiae* s.s. from Uganda and to deltamethrin in Uganda and Kenya [26]. Furthermore, this mutation has been shown to be highly diagnostic of extreme pyrethroid resistance, with survivors of two hour deltamethrin exposure significantly more likely to have L43F-CYP4J5, compared to those killed within one hour [26]. L43F-CYP4J5 lies within the 2La+^a^ inversion in *An. gambiae* s.l., which has previously been correlated with aspects of vector bionomics and competence, including adaptation to aridity or humidity [39], biting and resting behaviour [40] and susceptibility to *P. falciparum* infection [41]. In this study, evidence for ongoing selection of this inversion was apparent in Tchonka, which may in part explain the deviations from Hardy–Weinberg equilibrium observed for L43F-CYP4J5 in this site, but not in Tushunguti or Kibali. Importantly, L43F-CYP4J5 may warrant further monitoring as a potential predictor of extreme pyrethroid resistance in the DRC.

This study also presents the first report of G119S-*ace1* from all study sites at low frequencies (0.11–0.19), which did not appear to be under local selection at the time of sample collection. Duplication of the G119S-*ace1* mutation can enhance resistance to organophosphates and carbamates [42], including pirimiphos-methyl which is widely used in IRS campaigns [43], by reducing sensitivity to the neurotransmitter acetylcholinesterase [44]. In these study sites, limited insecticide spraying operations have been conducted, except in Kibali in the gold mining region. It is difficult to infer the emergence of G119S-*ace1* in direct response to public health insecticides; rather this may have been selected under the pressure of widespread, unregulated agricultural pesticide use [45, 46]. Finally in *An. gambiae* s.l. the presence of a triple mutant haplotype (a non-synonymous SNP in CYP6P4, an upstream insertion of a partial Zanzibar-like TE and duplication of the CYP6AA1 gene), associated with high levels of deltamethrin resistance [27], was identified at high frequencies across these study sites and represents an additional promising diagnostic marker for future surveillance of pyrethroid resistance.

Previous studies in the DRC have reported overexpression of CYP6P9a and CYP6P9b in *An. funestus* s.l. as mechanisms of pyrethroid and DDT resistance in Kinshasa and Haut-Uélé provinces [12, 13]. The presence of *cis*-regulatory polymorphisms in CYP6P9a and CYP6P9b, which drive overexpression, have been correlated with reduced efficacy of deltamethrin ITNs [18, 19]. Using two DNA-based assays, moderate to high frequencies of both resistance alleles were identified, with potential evidence for ongoing selection of CYP6P9a in Tchonka and CYP6P9b in Tushunguti. For both *An. gambiae* s.l. and *An. funestus* s.l., the impact of some of these mutations on intervention effectiveness, coupled with recent data demonstrating partial restoration of pyrethroid susceptibility following PBO pre-exposure in bioassays [8, 10], improved killing of populations containing triple mutants with PBO-ITNs [27] and high mortality to the putative diagnostic doses of chlorfenapyr [47], strongly support targeted deployment of next-generation synergist- and dual-active ingredient ITNs to control pyrethroid-resistant vector populations in the DRC.

Consistent with other  reports from the DRC, very high *P. falciparum* infection rates were detected, which were comparable between both *An. gambiae* s.l. and *An. funestus* s.l. across study sites (10.94–11.04%); *P. falciparum* infection prevalence was twice as high for both species in Tchonka compared to Tushunguti. While this study used PCR to assess overall vector infection rate, comparably high sporozoite rates in *An. gambiae* s.l., measured by ELISA, have been previously described from nearby Kashuga, Nord-Kivu (13.9%) [32]. While malaria transmission is known to be highly heterogeneous across the country, these observations of high *Plasmodium* infection levels in pyrethroid-resistant vector populations, which may be less responsive to standard ITNs, are of considerable concern.

## Conclusions

Real-time tracking of insecticide resistance is currently limited by the lack of diagnostic markers of intervention efficacy and difficulties dissecting the relative contributions of different mechanisms to resistance phenotype, particularly those involved in metabolic resistance. This study screened the most up-to-date panel of DNA-based resistance markers for target site and metabolic resistance in *An. gambiae* s.l. and *An. funestus* s.l. populations from the eastern DRC, where resistance data is lacking. Several new candidate markers (L43F-CYP4J5, G119S-*ace1*, the triple mutant: CYP6P4-I236M-Zanzibar-like TE-CYP6AA1 duplication, CYP6P9a and CYP6P9b) were identified, which are diagnostic of resistance to major insecticide classes used for malaria vector control and/or reduced pyrethroid ITN efficacy, and warrant future, larger-scale monitoring in the DRC to inform vector control decisions by the NMCP.

## Supplementary Information

**Additional file 1: TableS1.** Summary of all published insecticide resistance mechanism data from the DRC.

## Data Availability

Not applicable.

## Abbreviations

*Ace1*: Acetylcholinesterase
ANC: Antenatal care
BP: Bbase pair
*Cox-1*: Cytochrome c oxidase subunit 1
CDC: Centers for Disease Control and Prevention
CNV: Copy number variant
CYP450: Cytochrome-dependent monooxygenase 450
DDT: Dichlorodiphenyltrichloroethane
DHS: Demographic and Health surveys
DRC: Democratic Republic of the Congo
EPI: Expanded programme on immunization
GST: Glutathione-*s*-transferase
IRB: Institutional review board
IRS: Indoor residual spraying
ITN: Insecticide-treated net
*Kdr*: Knock-down resistance
LD: Linkage disequilibrium
LNA: Locked-nucleic acid
LSHTM: London School of Hygiene and Tropical Medicine
NMCP: National Malaria Control Programme
NTC: No template control
OR: Odds ratio
PBO: Piperonyl butoxide
*rdl*: Resistance to dieldrin
SNP: Single-nucleotide polymorphism
TE: Transposable element
VGSC: Voltage-gated sodium channel
